# The Mediterranean deep-water kelp *Laminaria rodriguezii* is an endangered species in the Adriatic Sea

**DOI:** 10.1007/s00227-016-2821-2

**Published:** 2016-03-14

**Authors:** Ante Žuljević, Akira F. Peters, Vedran Nikolić, Boris Antolić, Marija Despalatović, Ivan Cvitković, Igor Isajlović, Hrvoje Mihanović, Slavica Matijević, Dawn M. Shewring, Simonepietro Canese, Christos Katsaros, Frithjof C. Küpper

**Affiliations:** Institute of Oceanography and Fisheries, Šet. I. Meštrovića 63, 21000 Split, Croatia; Bezhin Rosko, 40 rue des pêcheurs, 29250 Santec, Brittany, France; Oceanlab, University of Aberdeen, Newburgh, AB41 6AA Scotland, UK; Institute for Environmental Protection and Research ISPRA, Via Vitaliano Brancati 48, Rome, 00144 Italy; Department of Botany, Faculty of Biology, University of Athens, Panepistimiopolis, Athens, 157 84 Greece

**Keywords:** Endangered algae, Circalittoral, cox1, Rubisco spacer, ITS, Kelp, Bottom trawling, IUCN criteria

## Abstract

Deep-water kelps are little-known large brown algae occurring close to the lower limit of photosynthetic life in the sea. This study compares historical and recent records of the deep-water Mediterranean kelp *Laminaria rodriguezii* in the Adriatic Sea. Historical records include data from herbarium collections and trawling fishery expeditions in the mid-twentieth century, while recent data comprise records of the last 17 years from MEDITS expeditions, ROV surveys of historical kelp locations, benthic surveys and records by fishermen. Altogether, these findings demonstrate that the Adriatic population of *L. rodriguezii* has suffered a decline of more than 85 % of its historical range and is now present only around the small offshore island of Palagruža. Bottom trawling activities are presumably responsible for the disappearance elsewhere. We propose to classify *L. rodriguezii* as “Endangered” in the Adriatic Sea under IUCN criteria B1ab(i,iii,iv), ver 3.1. Oceanographic characteristics of the habitat suggest that besides high water transparency, presence of North Adriatic Dense Water with both strong currents and stable low temperatures of around 14 °C are essential oceanographic factors for the development of *L. rodriguezii* in the Central Adriatic. The origin of cold water thus differs from that at upwelling sites permitting populations of tropical deep-water kelps. The phylogenetic position of *L. rodriguezii* is so far unknown. DNA sequences from nuclear and cytoplasmic markers of two thalli from Croatia and the western Mediterranean confirmed that *L. rodriguezii* is a member of the Laminariaceae and most closely related to *L. ochroleuca*, *L. pallida* and the Brazilian deep-water kelp *L.* *abyssalis*.

**Electronic supplementary material:**

The online version of this article (doi:10.1007/s00227-016-2821-2) contains supplementary material, which is available to authorized users.

## Introduction

Surface waters of the Mediterranean Sea show strong seasonal temperature fluctuations. While winter and spring temperatures in the range of 12–17 °C are similar to neighbouring areas of the North Atlantic, summer temperatures of 20–28 °C are closer to the temperatures of tropical seas (e.g. Coll et al. 2010). Consequently, biota living in these surface waters are subject to a strong, temperature-driven selective pressure, excluding organisms from both cold-temperate and tropical regions.

Kelps (Laminariales) are generally considered as macroalgae with temperate and cold-water affinities (Steneck et al. 2002). Their distribution in lower latitudes is limited to waters rarely exceeding 20 °C; furthermore, many species require lower winter temperatures for gametogenesis (Lüning 1980). In cases of strong stratification and high water transparency, kelp communities may occur in a cold-water layer receiving enough sunlight for photosynthesis beneath a constantly or seasonally warmer surface layer. Currently known deep-water kelps include *Laminaria rodriguezii* Bornet in the Mediterranean (Feldmann 1934), *L. abyssalis* A. B. Joly et E. C. Oliveira in Brazil (Joly and Oliveira 1967), *L.* *philippinensis* J. E. Petrov & M. V. Suchovejeva in the Philippines (Petrov et al. 1973) and *Eisenia galapagensis* W. R. Taylor in the Galapagos Islands (Graham et al. 2007).

In contrast to their well-studied relatives in shallow waters, deep-water kelp taxa remain poorly known in key aspects of their biology (Bartsch et al. 2008). *Laminaria abyssalis* is the best studied deep-water kelp species (Yoneshigue-Valentin 1990; Braga and Yoneshigue-Valentin 1996; Rodrigues et al. 2000, 2002; Yoneshigue-Valentin et al. 2003; Romanos et al. 2011; Marins et al. 2012). In contrast, only the original description (Petrov et al. 1973) exists for *L. philippinensis*.

*Laminaria rodriguezii* is an endemic deep-water Mediterranean macroalga consisting of a branched holdfast, a stipe and an undivided blade of up to 150 × 30 cm in size (Beck 1896; Hamel 1931–1939). It is able to produce adventive blades vegetatively on stolons (Huvé 1955), which is a rare feature in the Laminariales and shared in the genus *Laminaria* only with *L.* *sinclairii* (Harvey) Farlow, Anderson & Eaton from the northeast Pacific (Demes and Graham 2011). Originally described by Bornet (1888), it is scattered in the Western Mediterranean and Adriatic Sea. The main populations occur around the Balearic Islands (Joher et al. 2012) and on seamounts in the Tyrrhenian Sea (Giaccone 1967; Bo et al. 2011). The species occurs in areas of very specific biotic and abiotic parameters, which are still only partly understood. It was mainly reported from depths >70 m, seldom found between 50 and 70 m, and exceptionally less deep on seamounts or in upwelling systems (30 m at Galite Island area in Tunisia; Ballesteros 2006). The maximum depth, 260 m, was recorded in the Adriatic Sea (Ercegović 1960). *L.* *rodriguezii* occurs on loose-lying deep-water coralligenous beds (Ballesteros 2006) and may cover sea mounts, slopes and rocky ledges of offshore islands with prevailing highly transparent waters (Feldmann 1934; Giaccone 1969; Fredj 1972; Barcelo 1985; Bo et al. 2011). The upper limit possibly depends on temperature, while the lower limit is presumably determined by light availability (Joher et al. 2012). Since *L. rodriguezii* also lives in bottom trawling areas, there is concern on its conservation status (UNEP/IUCN/GISPosidonie 1990).

In the Adriatic Sea, *L. rodriguezii* is only reported from the Central Adriatic (mainly in Croatian, occasionally in international waters). First detailed knowledge of its distribution in the region was obtained during the Hvar fisheries biology expedition in 1948–1949 (Ercegović 1960), which was conducted to gather data on commercial trawl resources after several years of suspension of fishing during the Second World War. This dataset is a baseline (*zero state*) dataset of trawling bottom species till 400 m depth (Vrgoč et al. 2004). The expedition also collected data on bycatch including benthic seaweeds. *L.* *rodriguezii* was found in the areas of Jabuka Pit (henceforth referred to as “Jabuka”), Biševo Island (“Biševo”) and Palagruža Island (“Palagruža”). Between 1956 and 1961, further fisheries expeditions also recorded data on deep-water algae (Gamulin–Brida 1965). During fisheries expeditions in the following three decades, data on *L. rodriguezii* were not collected.

Since 1996, annual fishery surveys (Mediterranean International Bottom Trawl Suveys, MEDITS) using trawl nets have been conducted, covering transects similar to the Hvar expedition (Bertrand et al. 2002). This allowed a comparison of historical fisheries data with new records. Within the framework of the project “Brown algal biodiversity and ecology in the Eastern Mediterranean Sea”, we have recently conducted deep-water transects in the areas of the Adriatic Sea where *L. rodriguezii* was recorded in the 1950s and 1960s, using a ship-deployed remotely-operated vehicle (ROV). The results of these transects, together with the biological surveys performed from the mid-1990s, combined with information provided by professional fishermen, give an overview of the recent populations of *L. rodriguezii* in the Adriatic Sea and allow to evaluate its distribution and conservation status.

Little is known about the biology of *L. rodriguezii*, and in particular, there is no physiological knowledge about this species, which is without doubt because of the difficulty to collect live material. Also, the phylogenetic position of *L. rodriguezii* has not been clarified so far, with no sequence data having been reported. Using two thalli from the Adriatic and the western Mediterranean, as well as samples of putative relatives, we have generated sequences of nuclear and cytoplasmic markers, which suggest a close relationship with other Atlantic members of *Laminaria*.

## Methods

### Historical and spatial distribution of *Laminaria rodriguezii*

Data on the historical and current distribution of *L. rodriguezii* in the Adriatic were obtained from published and unpublished records. Unpublished data included specimens of *L.* *rodriguezii* housed in the main European and all Croatian herbaria, data collected during biological field surveys coordinated by the Institute of Oceanography and Fisheries in Split (IOF), and data obtained from fishermen since 2011.

**Fig. 1:**
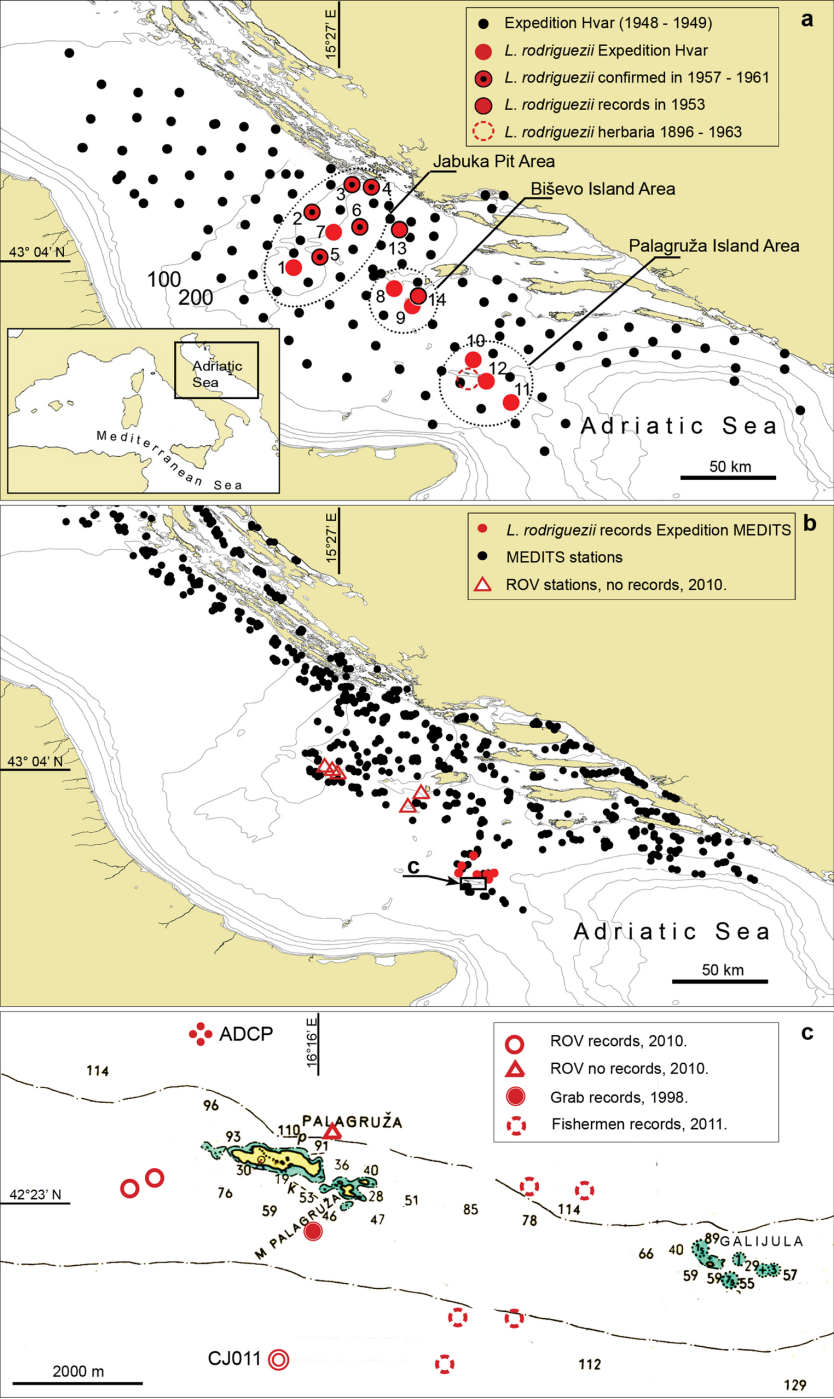
Historical and current distributions of *Laminaria rodriguezii* in the Adriatic Sea. **a** Hvar expedition stations (Ercegović 1960) and records of *L. rodriguezii* during that and two subsequent fishery expeditions in 1953 (Grubišić and Gospodnetić 1955) and 1957–1961 (Gamulin–Brida 1965). Including herbarium records until the 1960s (Palagruža area), we consider this distribution as historical or *zero state* of *L. rodriguezii* distribution in the Adriatic Sea. **b**, **c** Records of *L. rodriguezii* in the Adriatic Sea since 1997. **b** Stations of ROV and MEDITS expeditions and records of *L. rodriguezii.* **c** Location where *L. rodriguezii* thalli were collected close to Palagruža Island and positions of ADCP and permanent oceanographic station CJ011. The *dashed line* indicates the 100 m depth limit

ROV surveys were conducted by IOF in May 2010 on eight transects in the areas with historical records of *L. rodriguezii* (Fig. 1b, c). ROVs were equipped with robot arms, enabling in situ collections of specimens.

To evaluate the extinction risk of *L. rodriguezii* in the Adriatic Sea as one of the subregions of the Mediterranean Sea, we used the IUCN Red List criteria and guidelines for regional level (IUCN 2012).

### Oceanography

Oceanographic data (bottom water temperature, salinity, nutrients and surface transparency) of the area in which *L. rodriguezii* has been found during the last 17 years (Palagruža) were obtained from the permanent oceanographic station (Supporting Information).

Current measurements were conducted for 9 months at an ADCP station located 2.6 km to the northwest of Palagruža (Fig. 1c) (Supporting Information).

### Molecular phylogeny

DNA extraction was carried out on a specimen of *L. rodriguezii* collected near Palagruža (Adriatic Sea) and a herbarium specimen from Hecate Reef, Skerki Bank (international waters between Sicily and Tunisia), as well as on samples of *L.* *ochroleuca* Bachelot de la Pylaie, *L.* *pallida* Greville and *L.* *abyssalis* (Table S1; Supporting Information). *L.* *ochroleuca* is distributed in the NE Atlantic and in the western Mediterranean, including in deep water (Templado et al. 2006), *L. pallida* occurs in the upper subtidal in SW Africa and some temperate islands of the southern hemisphere (Stegenga et al. 1997), and *L.* *abyssalis* in deep water off Brazil (Marins et al. 2012). Molecular work on the herbarium specimen from Hecate Reef was realized in a different laboratory and years before studying the other samples.

Polymerase chain reactions (PCR) were performed using specific primers for nuclear ribosomal DNA (SSU, ITS), the plastid-encoded *rbc*L and Rubisco spacer, and the mitochondrion-encoded genes *cox*1, *cox*3, and *nad*6. Primers and PCR conditions used are provided in the appendix. The new sequences were compared with published sequences using BLAST (Altschul et al. 1997); ITS, Rubisco spacer and 5′-COI sequences were manually aligned in Se-Al™ v2.0a11 (Sequencing Alignment Editor Version 2.0 alpha 11; http://tree.bio.ed.ac.uk/software/seal/) with published sequences. Neighbour-joining distance analyses using Kimura-2-parameter distances, maximum parsimony and maximum likelihood analyses were performed in PAUP (Swofford 2002).

## Results

### Historical and recent records of *Laminaria rodriguezii* in the Adriatic Sea

Historical data were compiled from herbarium samples, from the Hvar expedition (Ercegović 1960) and from fishery and biocenology expeditions in the 1950s and early 1960s (Grubišić and Gospodnetić 1955; Gamulin–Brida 1965). They provided information about the historical distribution of *L. rodriguezii* (Figs. 1a, S1A, Tables 1, S2).

**Table 1:** Records of *Laminaria rodriguezii* in the Adriatic Sea until 1961, based on bottom trawling records of the Hvar Expedition (Ercegović 1960) and fishery—biocenology expeditions in the Areas of Jabuka (J), Biševo (B) and Palagruža (P) in 1953 (Grubišić and Gospodnetić 1955) and 1957–1961 (Gamulin–Brida 1965)

Location (Fig. 1a)	Area	Reported depth (m)	*L. rodriguezii* records			Substratum	T °C	Sal	Additional species
			Exp Hvar 1948–1949	Exp 1957–1961	Exp 1953				
1	J	256–262	+	n.v.	n.v.	Clay	10.2	38.4	*Sargassum vulgare*, *Halarachnion spathulatum* f*. luxurians*
2	J	188	+	+	n.v.	Silty clay	11.4	38.4	*Cystoseira foeniculacea* ssp*. latiramosa*, *Halarachnion spathulatum* f. *luxurians*
3	J	181	+	+	n.v.	Silty clay	10.4	38.3	*Dictyota dichotoma, Cystoseira foeniculacea* ssp*. latiramosa*, *Sargassum hornschuchii*, *Halarachnion spathulatum* f. *luxurians, Polysiphonia* sp.
4	J	168	+	+	n.v.	Silty clay	–	–	*Cystoseira foeniculacea* ssp*. latiramosa*
5	J	188–192	+	+	n.v.	Silty clay	12.2	38.4	*Halarachnion spathulatum* f*. luxurians*
6	J	160–170	+	+	n.v.	Silty clay	13.0	38.6	*Sargassum vulgare*, *Halarachnion spathulatum* f*. luxurians*
7	J	150	+	Not found	n.v.	Silt	11.7	38.4	*Cystoseira foeniculacea* ssp*. latiramosa*, *Halarachnion spathulatum* f. *luxurians*
8	B	126–130	+	n.v.	n.v.	Sandy clay	13.1	38.6	absent
9	B	158	+	n.v.	n.v.	Silt	12.4	38.4	*Halarachnion spathulatum* f. *luxurians*
10	P	130–132	+	n.v.	n.v.	Sand	12.6	38.5	absent
11	P	128	+	n.v.	n.v.	Silty clay	12.2	38.6	absent
12	P	120	+	n.v.	n.v.	Silty clay	–	–	*Carpomitra costata*, *Desmarestia adriatica*, *Rytiphlaea tinctoria*
13	J	118–128	n.v.	+	n.v.	–	–	–	*Cystoseira foeniculacea* ssp*. latiramosa*, *Sargassum vulgare*, *Sargassum hornschuchii*, *Sebdenia dichotoma*, *Halarachnion spathulatum* f. *luxurians*
14	B	80–90	n.v.	n.v.	+	Rocky	–	–	–

The expedition of 1957–1961 covered six identical stations where *L. rodriguezii* was recorded during Hvar expedition. For “Location” see Fig. 1a. Bottom temperature, salinity and additional macroalgal species were recorded during the Hvar expedition. Names of additional species (as reported by Ercegović 1960) have been updated according to current nomenclature (Guiry and Guiry 2015). “n.v.” not visited, “–” no data

#### Herbaria

Ten historical and two recent specimens of *L. rodriguezii* collected in the Adriatic Sea have been deposited in herbarium collections (Table S2). Eleven of them were collected from Palagruža (Fig. S1A) while for one there are no data on the collecting site.

#### Expedition Hvar, 1948–1949

Using trawl net and dredge, *L. rodriguezii* was collected on 12 different transects mostly in the Jabuka and Palagruža and one near Biševo (Fig. 1), at depths between 130 and 260 m (Table 1). The abundance of collected specimens was not provided.

#### Fishery and biocenology expeditions in 1950s and early 1960s

A fishery expedition in the area of Biševo in 1953 detected a shallower (93–65 m) community, “densely overgrown” with *L. rodriguezii* (Grubišić and Gospodnetić 1955; Table 1). During the biocenology expeditions performed in 1957–1961, the occurrence of *L. rodriguezii* was confirmed in Jabuka at 5 out of 6 locations where it had been found during the Hvar expedition, and collected at one new location at Jabuka (Fig. 1a; Table 1). During these expeditions, *L. rodriguezii* was categorized in the lowest abundance category with an average number of specimens of less than 10 per transect.

#### Unpublished records and expeditions MEDITS from 1998 to 2014

In 1998 a thallus of *L. rodriguezii* was collected near Palagruža Island by a grab at 118 m depth. Since 2002, during 14 annual MEDITS fisheries expeditions (including data from 2015) with more than 225 transects inside the documented historical distribution range of *L. rodriguezii*, the alga was collected on a total of seven occasions during bottom trawling transects between 155 and 200 m, all in the area of Palagruža (Fig. 1b; Table 2). On these seven occasions, *L.* *rodriguezii* was very rare and present only in fragments (Fig. S1B). MEDITS expeditions (Bertrand et al. 2002) have covered 835 transects in Croatian waters since 2002.

**Table 2:** Recent records of *Laminaria rodriguezii* in the Adriatic Sea (1998–2013)

Code in Fig. 1	Year	Depth (m)	Sediment type
Grab sampling	1998	118	Sandy bottom with calcareous Rhodophyta
MEDITS	2002	173–200	Muddy bottom
MEDITS	2002	172–183	Muddy bottom
MEDITS	2010	176	Sandy–muddy bottom
ROV	2010	90	Sandy bottom with calcareous Rhodophyta
MEDITS	2011	178	Muddy bottom
Fishermen’s records	2011	90–110	Not determined
MEDITS	2013	172	Muddy bottom
MEDITS	2013	182	Muddy bottom
MEDITS	2015	155–166	Sandy–muddy bottom

All were in the Palagruža area (Fig. 1b, c). Prevailing sediment type on MEDITS locations is defined indirectly on the basis of collected benthic invertebrates. Depths recorded by MEDITS expeditions must be considered with caution as these findings might represent drift material from shallower areas

#### Sampling by ROV

During a total of 14 ROV diving hours on coralligenous and detritic substrata between 60 and 130 m in the areas of Jabuka, Biševo and Palagruža in 2010, we encountered two thalli of *L. rodriguezii*, both close to Palagruža island at around 90 m depth (Figs. 1c, S1C, D; Table 3).

**Table 3:** Oceanographic parameters determined in the bottom layer (100 m) of the water column collected at oceanographic station CJ011 (Fig. 1c; bottom depth at 102 m) during 1994–2013

	SECCHI (m)	TEMP (°C)	SAL	O_2_ (ml/L)	O_2_ (%)	pH	TIN	N-ORG (mmol m^−3^)	HPO_4_^2−^(mmol m^−3^)	P-ORG (mmol m^−3^)	SIO_4_^4−^(mmol m^−3^)
Min	10	12.2	38.00	4.2	81.3	8.0	0.30	0.01	0.01	0.03	0.56
Max	30	15.2	39.01	6.1	105.9	8.3	6.20	11.15	0.29	0.36	6.25
Average	20	13.8	38.65	5.2	91.6	8.2	2.44	3.83	0.07	0.16	1.79
Median	21	13.8	38.69	5.1	91.5	8.2	2.32	3.42	0.06	0.14	1.55

This station is within the present distribution area of *Laminaria rodriguezii*

**Table 4:** Accessions of sequences, with new sequences in bold face

Sample	ITS	Rubisco spacer	5′-COI
*Laminaria rodriguezii* Croatia	**LN896341**	**LN896348**	**LN896336**
*Laminaria rodriguezii* Hecate	**LN896342**	**LN896349**	ND
*Laminaria abyssalis* Lü1291	**LN896343**	**LN896350**	**LN896337**
*Laminaria ochroleuca* BR93	**LN896344**	**LN896351**	**LN896338**
*Laminaria ochroleuca* GAL98	**LN896345**	**LN896352**	**LN896339**
*Laminaria pallida* NAM93	**LN896346**	**LN896353**	**LN896340**
*Laminaria pallida* SAF01	**LN896347**	**LN896354**	ND
*Laminaria digitata*	FJ042772	AY851559	AJ344328
*Laminaria digitata*	AF319014	AF318971	JN099683
*Laminaria hyperborea*	AY441771	AF318972	FJ409154
*Laminaria setchellii* Silva	AF319016	AF318973	GU097710
*Laminaria sinclairii*	AF319017	AY851558	KJ960264
*Laminaria ephemera* Setchell	FJ042733	AY851557	FJ409152
*Laminaria solidungula* J. Agardh	FJ042757	AY851556	FJ409161
*Laminaria yezoensis* Miyabe	FJ042749	AY851555	FJ409167
*Saccharina japonica* (Areschoug) Lane, Mayes, Druehl & Saunders	DQ143070	DQ143101	AP011493
*Saccharina sessilis* (C. Agardh) Kuntze	FJ042748	AY851553	FJ409207

The data for the different markers in the new sequences are from the same sample, whereas in the published sequences they are usually from different specimens of the same species

*ND* no data

#### Information from fishermen

Professional fishermen from the island of Lastovo, who perform trammel net fishing, provided personal accounts in 2011. They regularly collected *L.* *rodriguezii* as bycatch near Palagruža Island (Fig. 1c; Table 2) at depths between 90 and 110 m. Our confidence in these communications is high as the fishermen described *L.* *rodriguezii* as a “*flat brown seaweed with characteristic roots which firmly entangle into the net”.* Contrary to this, fishermen from trawling boats operating in the area of Jabuka and Biševo did not report any findings of *L. rodriguezii*.

### Oceanographic characteristics of the Palagruža area

Long-term data on oceanographic parameters are summarized in Table 3 and Fig. S4. The high-resolution temperature time series recorded by the ADCP at 163 m, less than 1 m above the sea bottom (Fig. S5) showed temperature oscillations between 10.67 (March 2012) and 14.63 °C (end of October 2012), which is in accordance with the long-term measurements (Fig. S4).

The distribution of near-bottom current speeds and directions at the ADCP station is shown as a rose plot in Fig. S6. Current direction was stable, the direction of the flow was in >65 % between east and southeast, indicating predominant outflow across the Palagruža Sill, towards the South Adriatic Pit. The strongest currents were also measured for these directions, with maximum speeds reaching 44 cm/s. Generally, near-bottom currents were relatively strong, with speeds larger than 10 and 20 cm/s encompassing more than 40 and 11 % of all measurements, respectively. They were particularly energetic between March and July 2012 (Fig. S7), when the dense water outflow was the strongest. The average bottom current speed during 9 months was 10.1 cm/s.

### Molecular systematic analyses

A total of 6000-bp DNA sequences for six markers (see “Methods” section; Table S3) were produced from the *L. rodriguezii* field specimen from Croatia. From the herbarium specimen of *L. rodriguezii* from the western Mediterranean, we generated a short sequence of 129 bp in the highly variable first part of ITS1. It was identical to the sequence from Croatia confirming conspecificity of the two samples. For the western Mediterranean specimen, also a Rubisco spacer sequence was produced which resembled the sequence from Croatia except for two substitutions. In addition, sequences of the highly variable “barcode” markers ITS, Rubisco spacer and 5′-COI were generated for *L. ochroleuca, L. pallida* and *L. abyssalis*. In the last species, the ITS sequence generated for our gametophyte culture was highly similar to four sequences recently published for field specimens from Brazil (Marins et al. 2012).

**Fig. 2:**
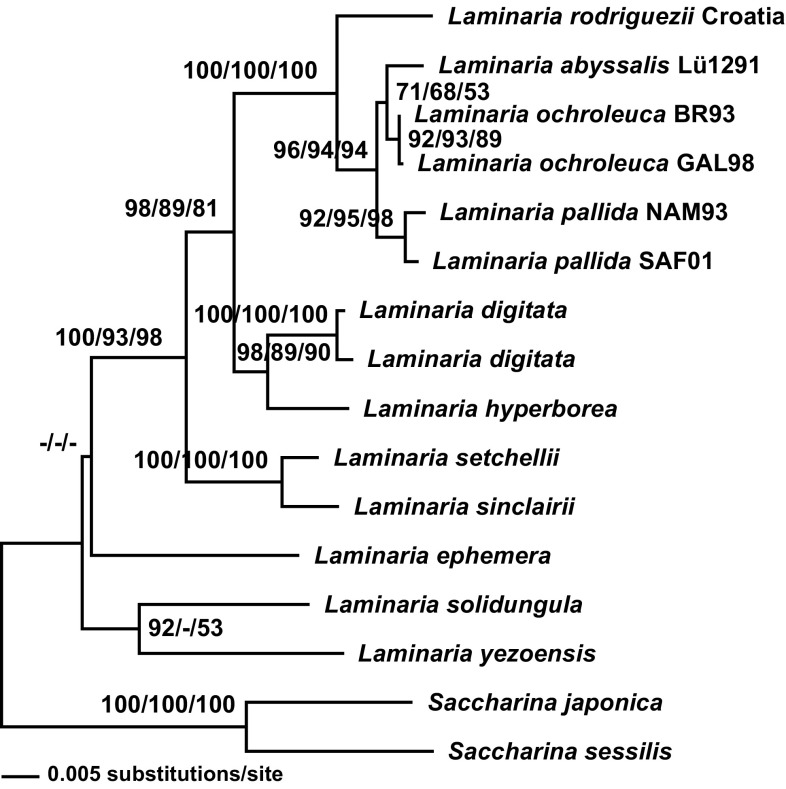
Phylogenetic tree of *Laminaria* spp. based on a neighbour-joining analysis of Kimura-2-parameter distances of concatenated entire ITS (763 bp), Rubisco spacer (553 bp) and 5′-COI (658 bp) sequences. Two species of *Saccharina* served as outgroup (Lane et al. 2006). Parsimony and maximum likelihood analyses of the same dataset, as well as analyses of data of the single markers, gave similar results, in most cases placing *L. rodriguezii* in a highly supported clade together with *L.* *ochroleuca, L. pallida* and *L. abyssalis*. Numbers at nodes indicate bootstrap support from 1000 resamplings for distance, parsimony and likelihood analyses, respectively. Accessions of sequences are provided in Table 4

In the sensitive, variable markers ITS and Rubisco spacer, *L. rodriguezii* showed most similarity with a cluster formed by *L.* *ochroleuca*, *L. pallida* and *L. abyssalis* (henceforth OPA clade), followed by *L.* *digitata* (Hudson) Lamouroux and *L. hyperborea* (Gunnerus) Foslie (Table S3). In the mitochondrial marker (5′-COI), genetic distances within the OPA cluster were ≤ 1.6 %, and *L.* *rodriguezii* was as divergent from the OPA clade (3.3–4.4 %) as from *L. digitata* (0.44 %) and *L.* *hyperborea* (0.39 %). Analyses of the combined dataset of ITS, Rubisco spacer and COI consistently placed *L.* *rodriguezii* in a highly supported clade together with the OPA clade (Fig. 2). In the more conservative *rbc*L and SSU, many species from the Laminariales showed highly similar sequences but phylogenetic analyses were not performed because sequences of the OPA clade were unavailable.

## Discussion

Historical data from herbarium samples collected from ca. 1896 until 1963 and from fishery expeditions (1949–1961) (Tables 1 and S2) indicated that *Laminaria rodriguezii* had three principal areas of distribution in the Adriatic Sea: Jabuka Pit and around the islands of Biševo and Palagruža, all of which are located in the central part of the Adriatic Sea (Fig. 1a). Fisheries expeditions from the 1960s until the early 2000s did not pay attention to *L.* *rodriguezii.* In the same period, commercial trawling effort increased, undoubtedly with consequences for benthic communities (Mannini and Massa 2000; Jukic-Peladic et al. 2001; Mannini et al. 2005). We thus consider the data on the distribution of *L. rodriguezii* in the Adriatic Sea until the early 1960s as historical range or *zero state*, similar to the *zero state* for bottom trawling of commercial fish species (Jukic-Peladic et al. 2001).

Recent data on the distribution of *L. rodriguezii* in the Adriatic Sea reveal that the distribution range of the species has drastically declined within the last 40 years (Fig. 1). Since 2000, the MEDITS project has surveyed almost the same transects on which *L. rodriguezii* was collected during the Hvar expedition in the late 1940s. Even though *L.* *rodriguezii* was one of the target species, the alga was recorded only at Palagruža. In the last 50 years, it has not been officially recorded in the area of Jabuka Pit where it was repeatedly found in the late 1940s and 1950s. Taking into account that the areas of Jabuka and Biševo have been well investigated recently through fisheries surveys, but also in particular during the deep-water brown algal expeditions using ROVs, it can be said with high confidence that *L. rodriguezii* is no longer present at Jabuka and Biševo. Consequently, the extent of occurrence (as defined by IUCN 2012) of this alga within the Adriatic Sea has shrunk by more than 85 %, from 8700 km^2^ (area covering all records) to 1200 km^2^ (historical and recent records in the Palagruža Island area).

There is no information about historical or recent population densities of *L. rodriguezii* around Palagruža Island. The latest information provided by fishermen operating in the area suggests that certain dense and healthy populations still exist in a spatially reduced area SE of Palagruža Island, while in the wider area of Palagruža the species is rare. Low abundance in a wider area of Palagruža is indicated by sporadic bycatch of the alga during the MEDITS project, with just a few fragments collected in the last 14 years (Fig. 1b, c; Table 2). Additional underwater ROV surveys would be required for more precise information on the condition of the populations at the sites suggested by fishermen.

As Jabuka Pit is one of the most important trawling areas in the Adriatic (UNEP/CBD/EBSA/WS 2014), the disappearance of *L. rodriguezii* is likely a result of bottom trawling activities. Mechanisms of impact would be direct, by removal of algae as bycatch, and indirect by stirring up sediments (Jones 1992; Palanques et al. 2001). In the Jabuka Pit, a layer of dense bottom water can in some years persist over longer periods (Artegiani et al. 2001). Resuspended sediments, which circulate inside the enclosed water mass within the depression, would possibly decrease light intensity under the light compensation point of the algae. It was pointed out in other parts of the Mediterranean that frequent trawling might influence *L. rodriguezii* (UNEP/IUCN/GISPosidonie 1990; Joher et al. 2012), but the disappearance of *L.* *rodriguezii* from Jabuka and Biševo is up to now the greatest reported decline of this species.

The area of Palagruža is not an intensive trawling area like Jabuka (IOF, unpublished data) but still has important commercial fishing activity. Moreover, the locations where *L.* *rodriguezii* was recently reported by fishermen, observed and collected by ROV and grab (Fig. 1c), correspond to an area where trawling is not allowed (minimum 2 nautical miles from the Island) and only small scale fishing like trammel nets is performed. In addition, strong bottom currents in the Palagruža may allow fast removal of fine sediment dispersed in bottom water due to bottom trawling, and possibly also clean the surface of thalli from deposited sediments.

The historical records of *L. rodriguezii* at Jabuka, at 150–260 m depth, were the deepest of that species in the entire Mediterranean. Such a depth is in the range of the deepest records of seaweeds worldwide (Littler et al. 1985; Markager and Sand-Jensen 1992). Sporadic findings of the alga in the Palagruža area during MEDITS surveys during the last 14 years were also in the depth range between 170 and 200 m. Collected specimens did not have holdfasts, thus it is not possible to conclude with certainty whether they were attached or just dispersed fragments. Bottom currents of 50 cm s^−1^ might be sufficient for proper fragment drifting. However, according to data provided from ADCP, bottom currents with E–SSE directions (Fig. S6) could not transport fragments from Palagruža Island to locations where *L. rodiguezi* was collected during MEDITS campaigns (Fig. 1b; 10–30 km in N–NE direction). Anyway, depths reported by earlier studies based on bottom trawling surveys must be considered with a certain caution. Without direct observation by ROV, exact data on light intensity on the bottom and ideally supporting, ecophysiological experiments, we cannot be sure that *L. rodriguezii* can develop at such extreme depth. The lower depth limits for kelp is assumed to be at 1 % of surface irradiance (Lüning and Dring 1979); however, this may not apply for *L.* *rodriguezii*. Secchi values measured in the last 15 years were around 20 m, with a maximum of 30 m. Such surface values of transparency only might be indicative and indeed reveal no information about the light intensity reaching the bottom. Giaccone (Giaccone 1967) noted how Secchi disks were visible up to 35 m near the island of Ustica (Italy) where *L. rodriguezii* populations occurred between 45 and 85 m depth. At 85 m, the seabed at Ustica was too steep and formed an environment with low luminosity that did not allow life of photosynthetic organisms. The locations where the alga was collected near Palagruža and historically at Jabuka are mostly flat, and therefore probably allow development of the alga at greater depth (no shading effect) even if the transparency is equal. Similar to the Palagruža site, costal detritic bottoms occur in Spain, where *L. rodriguezii* has been mainly reported between 70 and 80 m, with a maximum at 95 m (Joher et al. 2012; Sergi Joher Sais pers. comm.).

There is not much information on oceanographic conditions prevailing in the locations of other populations of *L. rodriguezii* in the Mediterranean. However, most reports suggest that favourable conditions include a hard coralligenous or rhodolithic substratum, dim light, highly transparent open sea water due to very low abundance of suspended particles, water temperatures permanently below 15 °C, and constant, mostly unidirectional weak to strong currents (Ercegović 1960; Giaccone 1967; UNEP/IUCN/GISPosidonie 1990; Bellan-Santini et al. 2002). This study presents for the first time precise long-term measurements of temperature, currents, salinity and nutrients in the bottom seawater in the documented distribution range of *L.* *rodriguezii*. Seawater temperature is almost constantly around 14 °C with temperature extremes slightly above 15 °C and below 12 °C being a very rare occurrence over 60 years of seasonal measurements (Fig. S4).

Current measurements from the ADCP station close to Palagruža showed that bottom currents were relatively strong and very stable. The strong bottom current in the area is the result of formation and sinking of cold and dense North Adriatic Dense Water (NAdDW) formed in the North Adriatic shelf and coastal area (Mihanović et al. 2013). One of the two major pathways of the NAdDW from the shallow northern Adriatic shelf and Jabuka Pit to the deep South Adriatic Pit is located to the north of Palagruža (Vilibić et al. 2004; Janeković et al. 2014). Predominant flow directions measured at the ADCP station were between east and southeast, corresponding to the northern pathway of the NAdDW outflow across the Palagruža Sill. The NAdDW formation event that occurred in January/February 2012 was exceptional and bottom density currents between March and June 2012 were particularly intense. Still, even after that period, weaker but persistent bottom currents, flowing mostly towards E–SE were observed in the area, till the end of measurements in December 2012 (Fig. S7). Both current and temperature measurements corroborate the global perception how persistent and stable currents and low temperatures play important roles in the formation of favourable condition for the development of *L. rodriguezii*. Therefore, NAdDW outflow with both strong currents and low and relatively stable temperatures is probably essential for the development of *L.* *rodriguezii* in the Central Adriatic. It thus appears that presence of *L. rodriguezii* in the Adriatic Sea coincides with oceanographic conditions that differ from the upwelling of cold waters at deep water kelp sites in tropical regions (Graham et al. 2007).

Total inorganic nitrogen, orthophosphate and orthosilicate concentrations in the bottom layer (Table 3) were generally higher than in the surface or middle layer of the water column (Vilibić et al. 2012), due to remineralization processes from the sediment or to advective mixing of water masses from the Mediterranean. Increased TIN concentrations are particularly expressed during the summer season (Barić et al. 2002), as a consequence of more intensive nitrogen flux from sediment that can be an additional source of nutrients facilitating algal growth.

*Laminaria rodriguezii* has a disjunct distribution within the Mediterranean Sea. The population in the Adriatic Sea, although genetically similar according to ITS sequences (see below), does not seem to be connected to the populations in the Western Mediterranean, since there are no intermediate populations known. Long distances from the next populations and adverse water mass circulation probably inhibit transport of any reproductive phase. Natural reintroduction in the case of total regional extinction in the Adriatic Sea is therefore not possible.

The IUCN Red List criteria are commonly used for evaluating the extinction risk of species (IUCN 2012). However, applying these to macroalgae is not simple and there are only a few seaweed species in the world on which they have been applied (Brodie et al. 2009; Phillips and Blackshaw 2011), basically due to lack of information on historical and recent distributions and the species’ biology and ecology. Except for the present comparison of historical and recent records, this also applies to *L. rodriguezii* in the Adriatic. There is still limited knowledge on its biology, population density and exact distribution as well as changes of the last two over exact periods of time, which are relevant criteria for IUCN evaluation. However, such data are clearly very difficult to obtain for such a relatively inaccessible species. In the light of the drastic decline of more than 85 % of the extent of occurrence in the Adriatic, which is estimated to be nowadays around 1200 km^2^, and its disappearance in two of three historical distribution areas (Jabuka Pit and Biševo Island area), as well as still ongoing, strong bottom trawling activities (UNEP/CBD/EBSA/WS 2014), we propose that *L.* *rodriquezii* should be classified as “Endangered” under IUCN criteria B1ab(i,iii,iv), ver 3.1.in the Adriatic Sea.

A management plan should therefore be developed in order to enable maintenance of *L.* *rodriguezii* in the area of Palagruža Island, its last refuge within the Adriatic. Similar measures were proposed for an area with *L. rodriguezii* populations in the Menorca Channel (Barbera et al. 2012). The decline of canopy-forming species, especially those in shallow waters, is an expanding, worldwide trend, largely due to different human activities while only sporadic local actions are taken to remedy this situation (e.g. Thibaut et al. 2015; Yesson et al. 2015, and references therein).

Further ROV expeditions should be organized with the objective of not destructively quantifying the abundance of *L.* *rodriguezii* and to establish detailed parameters for a further monitoring program. The greatest risk for the species appears to consist in continued bottom trawling. Temperature increases in Mediterranean deep water may represent another threat. The distribution of shallow water kelp species was reported to react rapidly to small changes in seawater temperatures, possibly due to temperature requirements for reproduction (Bartsch et al. 2013). On the other side, Yesson et al. (2015) showed that temperature alone can not account for long-term increases and decreases in abundance of the large brown seaweeds observed around the British Isles and suggested a combination of both physical and biological factors as drivers of these changes. Therefore, the possible future impact of seawater temperature increase on deepwater *L. rodriguezii* must remain speculative, as there are no ecophysiological data on growth and reproduction of this species. So far, long-term measurements of sea bottom water in the Palagruža area have not shown any statistically significant temperature increase (Vilibić et al. 2012).

Sequence similarity in a highly variable part of ITS and the Rubisco spacer between our Croatian and Italian samples of *L. rodriguezii* suggested that there may not be much genetic differentiation between individuals from the two sites. The sequences of all markers confirmed that *L. rodriguezii* belongs to the Laminariaceae, which is at present (Guiry and Guiry 2015) understood as a clade formed by the type *L. digitata* and about 20 other species, including all Atlantic kelps with digitate blades, such as *L.* *hyperborea*, *L.* *ochroleucaand L.* *pallida*. According to the sequences studied, particularly ITS, *L. rodriguezii* is closely related to a clade formed by *L.* *ochroleuca, L. pallida* and *L. abyssalis.* Our sequences suggest that adaptation to deep water in *L.* *rodriguezii* and *L.* *abyssalis* possibly evolved independently and that the two are different species, which is born out by the fact that the adventive blades characteristic for *L.* *rodriguezii* are absent in *L.* *abyssalis*. However, these are preliminary data from three markers and few individuals. A more comprehensive phylogeographic hypothesis for *L.* *rodriguezii* would require sequences for several markers from replicate individuals from different populations of the two deep-water species as well as of all Atlantic kelp taxa. It is unknown if *L. rodriguezii* can cross with the members of the AOP clade; cross-fertility has been shown experimentally for *L. pallida* and *L. abyssalis* (Dieck and De Oliveira 1993). It is urgent to isolate gametophyte cultures of *L.* *rodriguezii* in order to study genetics and physiology of this impressive species.

## Conclusion

Comparison of historical and recent data on the distribution *L. rodriguezii* showed that according to IUCN criteria, the extent of occurrence of this deep water species within the Adriatic Sea has been drastically reduced by more than 85 % (from 8700 to 1200 km^2^). The most probable reasons for its disappearance are direct and indirect impacts of trawling (physical collecting and decrease of water transparency). Taking into account the IUCN criteria, we propose that this species is classified as “Endangered” under IUCN criteria B1ab(i,iii,iv), ver 3.1. in the Adriatic Sea. A management plan should therefore be developed to enable maintenance of *L. rodriguezii* in the area of Palagruža Island, which according to the data presented here is its last refuge within the Adriatic and the East part of the Mediterranean Sea.

## Electronic supplementary material

Below is the link to the electronic supplementary material.

Supplementary material 1 (PDF 1450 kb)
