# Supplementary material for: The Mediterranean deep-water kelp Laminaria rodriguezii is an endangered species in the Adriatic Sea
Source: Mar Biol. 2016 Mar 14;163:69. doi: 10.1007/s00227-016-2821-2 (PMC4791464; doi:10.1007/s00227-016-2821-2)
Supplement: Supplementary file 1 — Supplementary material 1 (PDF 1450 kb) [file 227_2016_2821_MOESM1_ESM.pdf]

Electronic Supplementary Material

The Mediterranean Deep-Water Kelp *Laminaria rodriguezii* is an Endangered Species in the Adriatic Sea

Ante Žuljević[a], Akira F. Peters[b], Vedran Nikolić[a], Boris Antolić[a], Marija Despalatović[a], Ivan Cvitković[a], Igor Isajlović[a], Hrvoje Mihanović[a], Slavica Matijević[a], Dawn M. Shewring[c], Simonepietro Canese[d], Christos Katsaros[e], Frithjof C. Küpper[c*]

[a]Institute of Oceanography and Fisheries, Šet. I. Meštrovića 63, 21000 Split, Croatia

[b]Bezhin Rosko, 40 rue des pêcheurs, 29250 Santec, Brittany, France

[c]Oceanlab, University of Aberdeen, Newburgh, AB41 6AA, Scotland

[d]Institute for Environmental Protection and Research, ISPRA,Via Vitaliano Brancati 48, 00144 Rome, Italy

[e]Department of Botany, Faculty of Biology, University of Athens, Panepistimiopolis, Athens 157 84, Hellas (Greece)

*Corresponding author: Oceanlab, University of Aberdeen, Newburgh, AB41 6AA, Scotland; phone: +44-1224-274490; E-mail: < fkuepper@abdn.ac.uk >

Table S1. Biological material used to generate DNA sequences. Med: Mediterranean, Atl: Atlantic, SP: sporophyte, GAm: male gametophyte, ND: not determined. Herbarium abbreviations according to the Index Herbariorum (http://sweetgum.nybg.org/ih/).

Species	Specimen	Coordinates	Locality	Sea	Date	Depth (m)	Collector/ isolator	Generation	Material	Voucher
L. rodriguezii	NHMS001311	42° 23.16'N 16° 14.03'E	Palagruža (Croatia)	Adriatic	May 2010	90	S. Canese (ROV)	SP	Live field thallus	Specimen deposited in NHMS, accession NHMS001311 (Fig. S2)
L. rodriguezii	Hecate Reef, Skerki Bank	37°46.52'N 10°49.17'E	Hecate Reef, Skerki Bank (Int. Water)	W Med	Aug 1994	28	B.Schaffelke (SCUBA dive)	SP	Herbarium specimen	Specimen deposited in PC, accession PC0145176 (Fig. S3)
L. abyssalis	Brazil Lü1291	ND	Cabo Frio (Brazil)	SW Atl	≤1987	ND	E. Oliveira/ I. Bartsch	GAm	Culture	Culture maintained at Bezhin Rosko and AWI Bremerhaven
L. ochroleuca	LochBR93	ND	Roscoff (France)	NE Atl	1993	Drift	AF Peters	GAm	Culture	Culture maintained at Bezhin Rosko
L. ochroleuca	LochGAL98	ND	Camariñas (Spain)	NE Atl	1998	Upper subtidal	AF Peters	GAm	Culture	Culture maintained at Bezhin Rosko
L. pallida	LpalNAM93	ND	Swakopmund (Namibia)	SE Atl	1993	Drift	AF Peters	GAm	Culture	Culture maintained at Bezhin Rosko
L. pallida	LpalSAF01	ND	Cape Town (South Africa)	SE Atl	2001	Low intertidal	AF Peters	SP	Live field thallus	Culture maintained at Bezhin Rosko

Table S2. Specimens of *Laminaria rodriguezii* collected in the Adriatic Sea and deposited in herbarium collections. Herbarium abbreviations: ZA – Faculty of Science, Zagreb, Department of Botany; BM - Natural History Museum in London; PC - National Museum of Natural History (France); TSB - Università degli Studi di Trieste; IOF – Institute of Oceanography and Fisheries in Split (not in Index Herbariorum); NHMS - Natural History Museum in Split.

Herbarium	Collection code	Collector	Location	Depth [m]	Date
ZA	algal collection	F. Steindachner	Palagruža	-	≤1896
BM	BM000563293	F. Steindachner	Palagruža	-	≤1896
BM	BM000563292	F. Steindachner	Palagruža	-	≤1896
PC	MNHN-PC-PC0199776	F. Steindachner	Palagruža	-	≤1896
ZA	algal collection	J. Schiller	Palagruža	70 - 90	01 Sep 1912[1]
ZA	algal collection	J. Schiller	Adriatic Sea[2]	-	ND
TSB	I/1014	J. Schiller	Palagruža	70 - 90	01 Sep 1912
TSB	II/1016	Seefeldner et Schiffner	Palagruža	-	01 June 1913
IOF	F40_BK2	A. Ercegović	Palagruža	70	12 May 1963
NHMS	NHMS001311	S. Canese	Palagruža	90	May 2010

(1) Two specimens; (2) possibly also Palagruža.

Molecular methods

Polymerase Chain Reactions were carried out using primer pairs (Table S1) with an initial denaturation at 94˚C for 5 min, followed by 40 cycles of amplification consisting of denaturation at 94˚C for 30 sec, annealing and extension steps. The 40 cycles were followed by a final extension at 72°C for 5 min. PCR amplification was performed using BIOTAQ™ DNA Polymerase (5 units/µl; Bioline); each 25 µl reaction contained 2.5 µl of 10 X NH_4 buffer, 1 - 4 µl of $MgCl_2$ (50 mM), 1 µl of dNTP mix (2.5 mM each), 0.5 µl each of forward and reverse primers (10 µM), 14.75 – 17.75 µl of PCR grade H_2O, 0.25 µl of BIOTAQ™ DNA Polymerase and 1.5 µl of template DNA. PCR products were electrophoresed on 1.2 % (w/v) agarose gels, which were stained in GelRed nucleic acid stain; DNA bands were visualized using a gel imager. Products of interest were Sanger sequenced, employing the same primers as for PCR, by specialised companies (MWG Eurofins or Source Bioscience).

Table S3. Oligonucleotide primers for PCR and sequencing.

Primer name	Forward/ Reverse	Sequence 5'-3'	Annealing site	Reference
P1	F	GGAAGGAGAAGTCGTAACAAGG	near end of SSU	Tai et al. (2001)
KG4	R	CTTTTCCTCCGCTTAGTTATATG	near start of LSU	Lane et al. (2006)
K1R1	R	TTCAAAGTTTTGATGATT	in 5.8S SU	Lane et al. (2006)
117F	F	TTTCHACNAAYCAYAAAGATAT	cox1	Bittner et al. (2008)
784R	R	ACTTCDGGRTGDCCAAAAAACCA	cox1	Bittner et al. (2008)
789F	F	TNTAYCARCATTTATTTTGGTT	cox1	Silberfeld et al. (2010)
1387R	R	TCYGGNATACGNCGNGGCATACC	cox1	Silberfeld et al. (2010)
JO3CSF	F	GTACACACCGCCCGTCGCAC	in SSU	Peters and Burkhardt (1998)
AFP1F	F	GCGGAAGGATCATTACCGAA	end of SSU	Peters and Burkhardt (1998)
5.8S1R	R	TGATGATTCACTGGATTCTG	in 5.8S SU	Peters and Ramírez (2001)
5.8S1F	F	CAGAATCCAGTGAATCATCA	in 5.8S SU	same as before, reverse-complement
LSU58R	R	CTTCACTCGCCGTTACTGG	in SSU	Peters et al. (2000)
rbcL1273F	F	GTGCGACAGCTAACCGTG	in rbcL	unpublished
rbcS139R	R	AGACCCCATAATTCCCAATA	in rbcS	Peters and Ramírez (2001)

Figure S1. *Laminaria rodriguezii* records in the Palagruža Island area. A) Herbarium sheet of *L. rodriguezii* collected by J. Schiller in 1912 and deposited in ZA. B) Sample obtained in 2010 during MEDITS bottom trawl surveys at station 10. C - D) Two thalli of *L. rodriguezii* observed in 2010 by ROV at around 90 m depth.

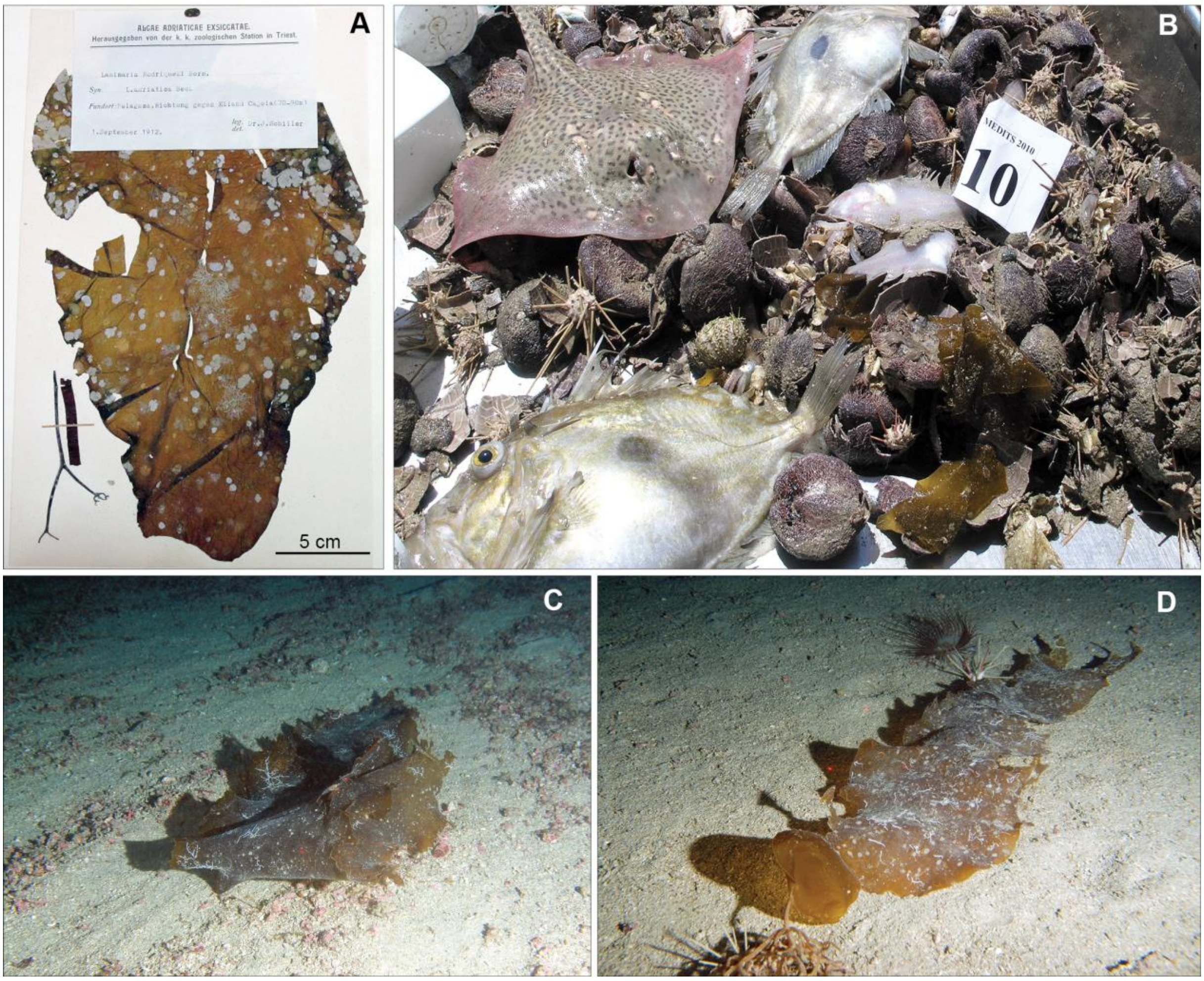

Figure S2. Specimen of *Laminaria rodriguezii* (NHMS001311) collected by ROV from the Adriatic Sea (Palagruža Island) (Fig. 1c) in May 2010 on 90 m depth, used for DNA extraction.

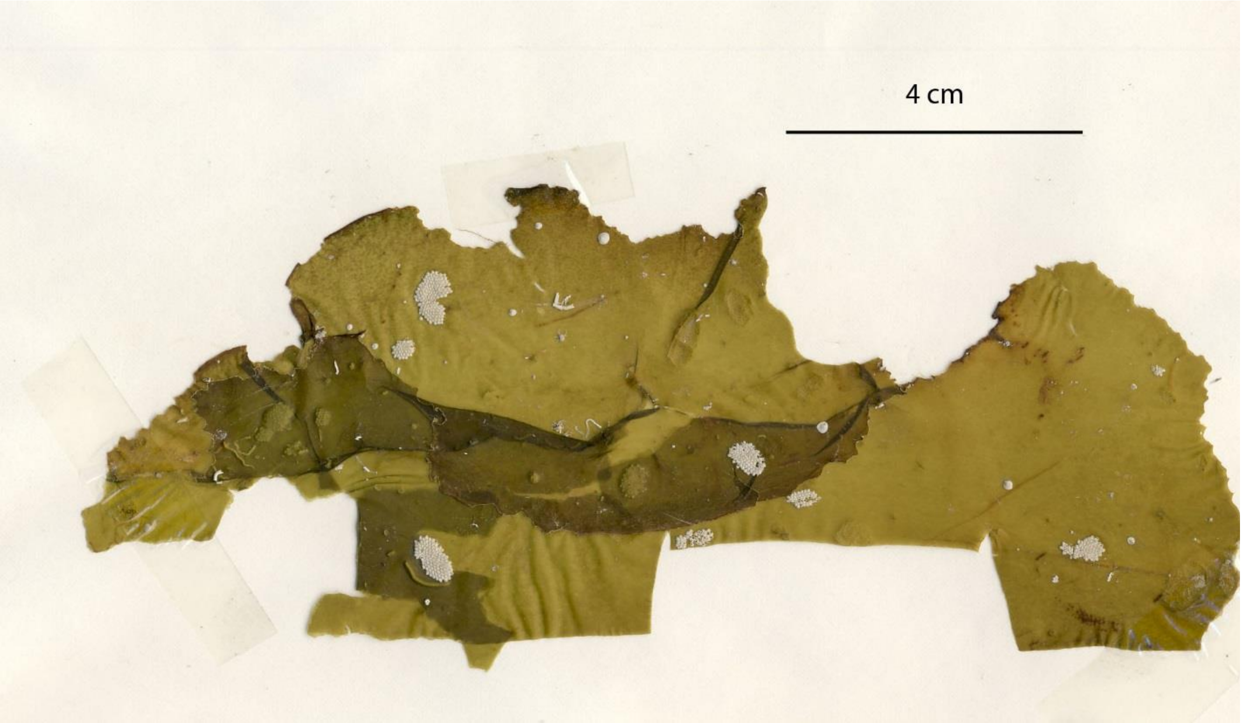

Figure S3. Specimen of *Laminaria rodriguezii* (PC0145176) from Hecate Reef (Western Mediterranean) collected by SCUBA diving at 28 m depth, used for DNA extraction.

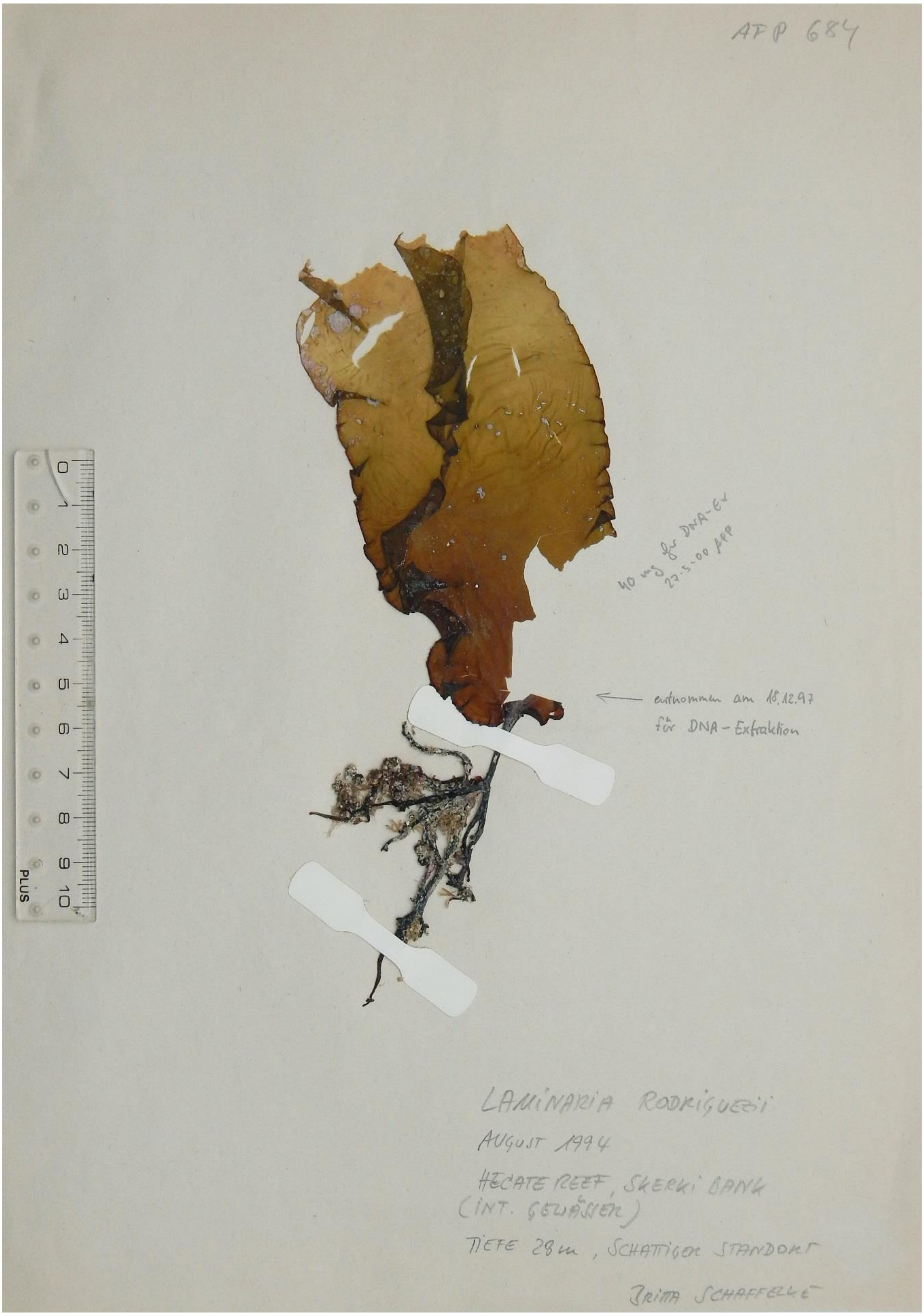

Figure S4. Seasonal variations of bottom (100 m deep, bottom depth on 102 m) temperature, salinity and transparency values between 1952 and 2013 at the oceanographic station CJ 011 near Palagruža island (see Fig. 1c for position).

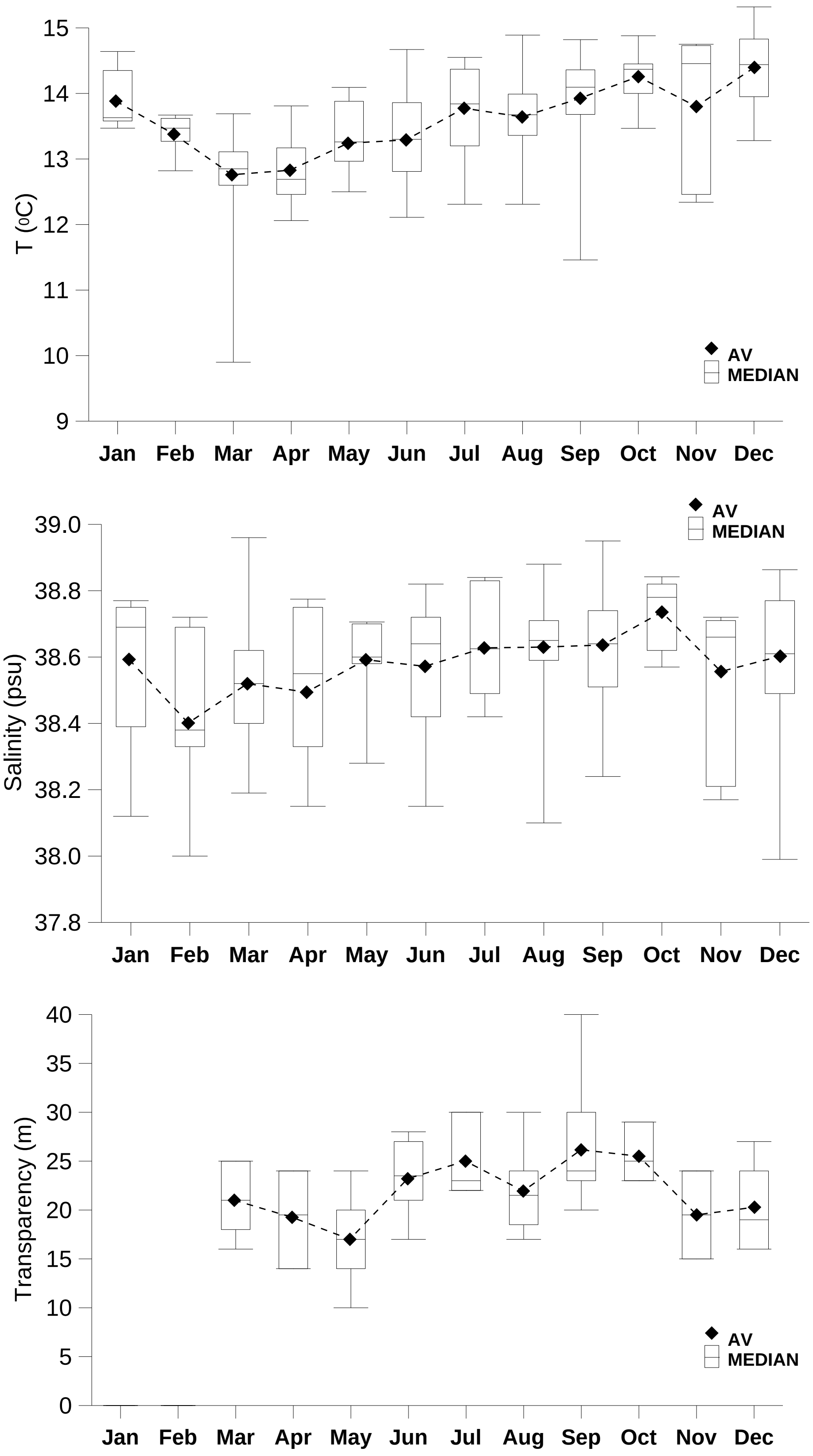

Figure S5. Seawater temperature recorded near the bottom of the ADCP station (at 163 m depth) between March and December 2012 (see Fig. 1c for the position of the station).

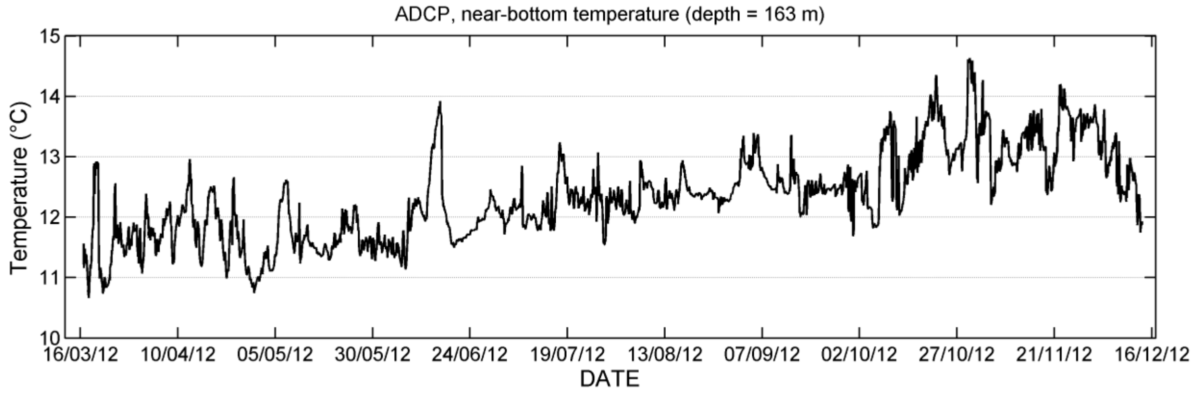

Figure S6. Rose plot for near-bottom currents (157.4 m) at the ADCP station (see Fig. 1c for the position of the station). The rose is drawn for 16 directions (using oceanographic convention, so that each of them denotes the direction where the current is flowing) while speeds are divided in classes with 5 cm/s range.

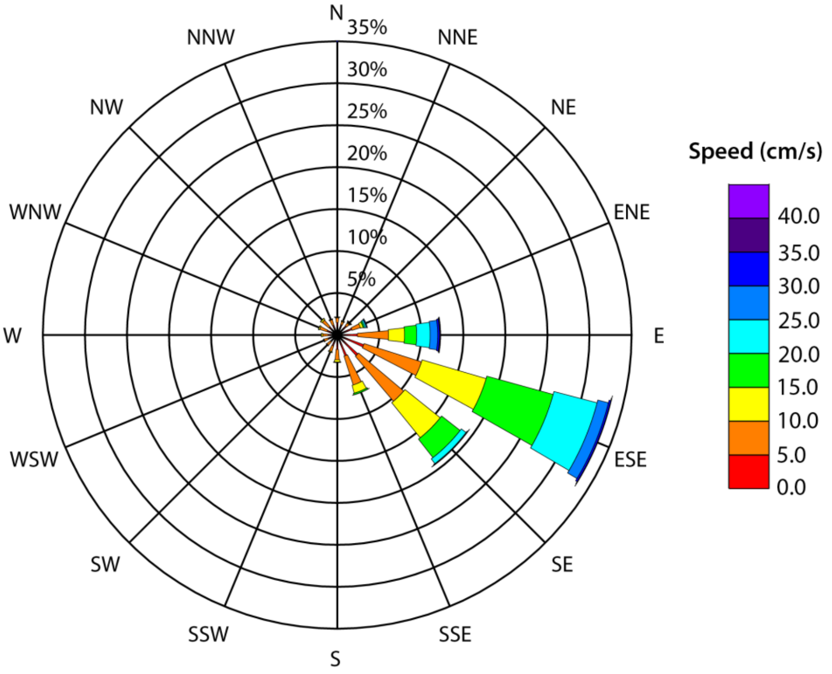

Figure S7. A) Hovmöller diagram of low-pass filtered (cut-off period of 33 h) current component projected in the direction of maximum variance of the bottom currents (angle of maximum variance equals 108.7° clockwise from the True North) from near the bottom (157.4 m) up to 80 m depth at the ADCP station (see Fig. 1c for the position of the station). B) The same as in A) except for the corresponding orthogonal (minor axis) component.

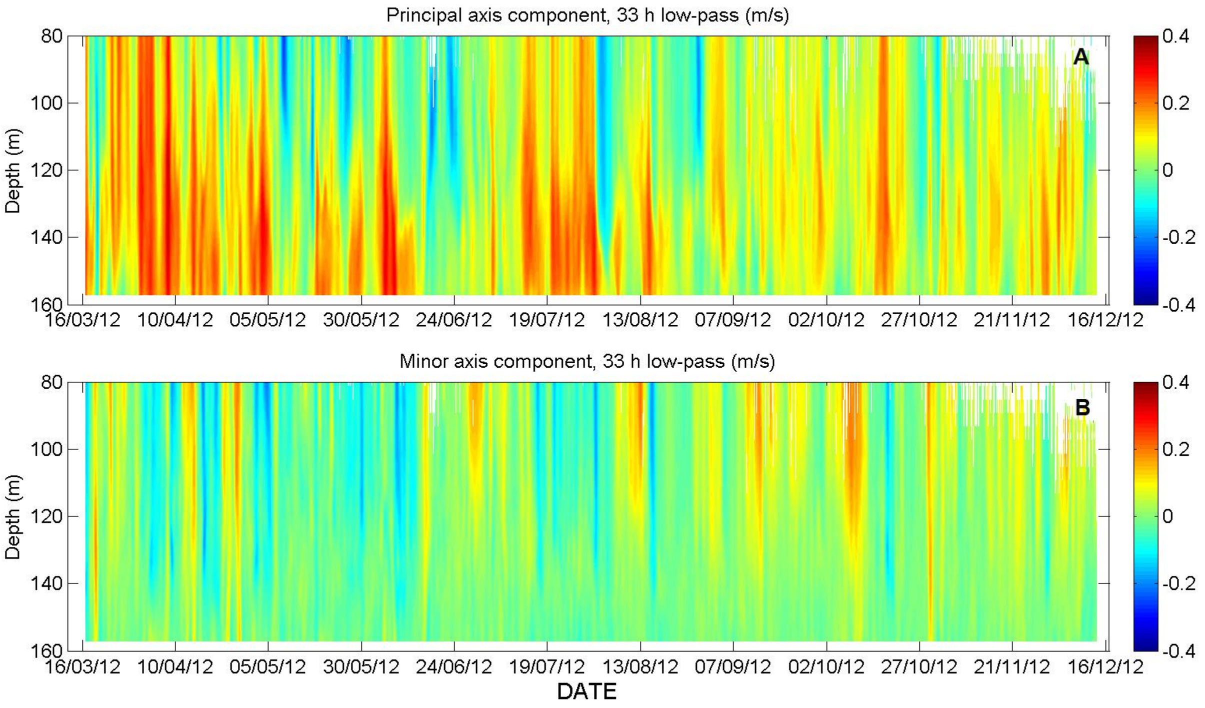

References

Bittner, L., Payri, C.E., Couloux, A., Cruaud, C., de Reviers, B., Rousseau, F., 2008. Molecular phylogeny of the Dictyotales and their position within the brown algae, based on nuclear, plastidial and mitochondrial sequence data. Mol. Phylogenet. Evol. 49: 211–226.

Lane, C.E., Saunders, G.W., 2005. Molecular investigation reveals epiphytic extrageneric kelp (Laminairales, Phaeophyceae) gametophytes on *Lessoniopsis littoralis* thalli. Bot. Mar. 48: 426–436.

Lane, C.E., Mayes, C., Druehl, L.D., Saunders, G.W., 2006. A multi-gene molecular investigation of the kelp (Laminariales, Phaeophyceae) supports substantial taxonomic re-organization. J. Phycol. 42: 493–512.

Peters, A.F. & Burkhardt, E. 1998. Systematic position of the kelp endophyte *Laminarionema elsbetiae* (Phaeophyceae, Ectocarpales *sensu lato*) inferred from nuclear ribosomal DNA sequences. Phycologia 37: 114-120.

Peters, A.F. & Ramírez, M.E. 2001. Molecular phylogeny of small brown algae, with special reference to the systematic position of *Caepidium antarcticum* (Adenocystaceae, Ectocarpales). Cryptogamie, Algologie 22: 187-200.

Peters, A.F., Ramírez, M.E. & Rülke, A. 2000. The phylogenetic position of the subantarctic marine macroalga *Desmarestia chordalis* (Phaeophyceae) inferred from nuclear ribosomal ITS sequences. Polar Biology 23: 95-99.

Silberfeld T., Leigh J.W., Verbruggen H., Cruaud C., Reviers, B. De & Rousseau F., 2010 - A multi-locus time-calibrated phylogeny of the brown algae (Heterokonta, Ochrophyta, Phaeophyceae): Investigating the evolutionary nature of the ‘‘brown algal crown radiation’’. Molecular Phylogenetics and Evolution 56: 659–674.

Tai, V., Lindstrom, S. C. & Saunders, G. W. 2001. Phylogeny of the Dumontiaceae (Gigartinales, Rhodophyta) and associated families based on SSU rDNA and internal transcribed spacer sequence data. J. Phycol. 37: 184–96.

White, T.J., Bruns, T.D., Lee, S.B. & Taylor, J.W. 1990. Analysis of phylogenetic relationships by amplification and direct sequencing of ribosomal genes. In: PCR protocols (Ed. by M.A. Innis, D.H. Geleand, J.J. Sninsky & T.J. White), p. 315-322. Academic Press, New York.
